# Outcome and survival of surgically treated acute subdural hematomas and postcraniotomy hematomas – A retrospective cohort study

**DOI:** 10.1016/j.bas.2023.102714

**Published:** 2023-11-22

**Authors:** Iiro Heino, Antti Sajanti, Seán B. Lyne, Janek Frantzén, Romuald Girard, Ying Cao, Joel F. Ritala, Ari J. Katila, Riikka S.K. Takala, Jussi P. Posti, Antti J. Saarinen, Santtu Hellström, Dan Laukka, Ilkka Saarenpää, Melissa Rahi, Olli Tenovuo, Jaakko Rinne, Janne Koskimäki

**Affiliations:** a Neurocenter, Department of Neurosurgery, Turku University Hospital and University of Turku, P.O. Box 52 (Hämeentie 11), FI-20521, Turku, Finland; b Department of Neurosurgery, Brigham and Women's Hospital, Harvard Medical School, Boston, MA, USA; c Neurovascular Surgery Program, Section of Neurosurgery, The University of Chicago Medicine and Biological Sciences, (5841 S. Maryland), Chicago, IL, 60637, USA; d Department of Radiation Oncology, Kansas University Medical Center, Kansas City, KS, 66160, USA; e Perioperative Services, Intensive Care Medicine and Pain Management, Turku University Hospital and University of Turku, P.O. Box 52 (Hämeentie 11), FI-20521, Turku, Finland; f Neurocenter, Turku Brain Injury Center, Turku University Hospital and University of Turku, P.O. Box 52 (Hämeentie 11), FI-20521, Turku, Finland; g Department of Clinical Neurosciences, University of Turku, P.O. Box 52 (Kiinamyllynkatu 4-8), FI-20520, Turku, Finland; h Department of Paediatric Orthopaedic Surgery, Turku University Hospital and University of Turku, P.O. Box 52 (Hämeentie 11), FI-20521, Turku, Finland

**Keywords:** Acute subdural hematoma, Postcraniotomy hematoma, Outcome, Survival, Surgery, Traumatic brain injury

## Abstract

**Background:**

The morbidity and mortality of acute subdural hematoma (aSDH) remains high. Several factors have been reported to affect the outcome and survival of these patients. In this study, we explored factors potentially associated with the outcome and survival of surgically treated acute subdural hematoma (aSDH), including postcraniotomy hematomas (PCHs).

**Methods:**

This retrospective cohort study was conducted in a single tertiary university hospital between 2008 and 2012 and all aSDH patients that underwent surgical intervention were included. A total of 132 cases were identified for collection of demographics, clinical, laboratory, and imaging data. Univariate and multivariable analyses were performed to assess factors associated with three-month Glasgow Outcome Scale (GOS) and survival at one- and five-year.

**Results:**

In this study, PCH (n = 14, 10.6%) was not associated with a worse outcome according to the 3- month GOS (p = 0.37) or one (p = 0.34) and five-year (p = 0.37) survival. The multivariable analysis showed that the volume of initial hematoma (p = 0.009) and Abbreviated Injury Scale score (p = 0.016) were independent predictors of the three-month GOS. Glasgow Coma Scale (GCS) score (p < 0.001 and p = 0.037) and age (p = 0.048 and p = 0.003) were predictors for one and five-year survival, while use of antiplatelet drug (p = 0.030), neuroworsening (p = 0.005) and smoking (p = 0.026) were significant factors impacting one year survival. In addition, blood alcohol level on admission was a predictor for five-year survival (p = 0.025).

**Conclusions:**

These elucidations underscore that, although PCHs are pertinent, a comprehensive appreciation of multifarious variables is indispensable in aSDH prognosis. These findings are observational, not causal. Expanded research endeavors are advocated to corroborate these insights.

## Highlights

• Larger hematoma & higher AIS score were independent predictors for worse 3-month functional outcome after surgery for aSDH.
• Lower GCS score at trauma scene & increased age were identified as predictors for worse survival after one & five years.
• Use of antiplatelet drugs was a predictor for worse survival after one year.
• Occurrence of postcraniotomy hematoma requiring reoperation was not associated with outcome or survival in this study.

## 1 Introduction

Acute subdural hematoma (aSDH) occurs in one third of severe traumatic brain injury (TBI) cases with a Glasgow Coma Scale (GCS) score of 8 or less (Chen et al., 2011; Godlewski et al., 2013). The concurrent incidence of other intracranial injuries such as intracerebral hematoma (ICH) and contusions are common, which creates challenges for the surgical and postoperative management of patients with TBI (Karibe et al., 2014; Bullock et al., 2006). While surgical intervention in aSDH is often a life-saving treatment, neurocritical care plays a vital role in the successful management of severe TBI. Despite advancements regarding the perioperative and postoperative treatment in aSDH, the mortality rate remains relatively high (Kotwica and Brzeziński, 1993; Massaro et al., 1996; Mathew et al., 1993; Seelig et al., 1981; Wilberger et al., 1991). Furthermore, the risk of mortality following severe head trauma remains increased in comparison to the general population for at least seven years (McMillan and Teasdale, 2007; Brown et al., 2004; Hukkelhoven et al., 2003; Harrison-Felix et al., 2004; Pentland et al., 2005; Signorini et al., 1999). Although, higher age is a risk factor for worse outcome in multiple studies, this does not preclude the younger population from substantial mortality risk (McMillan and Teasdale, 2007; Susman et al., 2002; Mosenthal et al., 2002). Prior research has demonstrated that in these aSDH patients age and GCS, as a surrogate for neurologic status, emerge as the current most important prognosticating factors (Kotwica and Brzeziński, 1993; Hatashita et al., 1993; Howard et al., 1989). Head computed tomography (CT) findings including hematoma thickness, hematoma volume, midline shift, and compression of basal cisterns also correlate with outcome after aSDH (Kotwica and Brzeziński, 1993; Howard et al., 1989; Zumkeller et al., 1996; Servadei et al., 2000). Finally, pupillary reactivity and pre-injury anticoagulation therapy have also been linked with outcome (Sakas et al., 1995; Won et al., 2017). Despite the recognition of these multifactorial risk factors, the medical and surgical therapy responding to them has left the rate of favorable outcome after aSDH regrettably low at 16–35% in recent studies (Lenzi et al., 2017; Chrastina et al., 2020).

A secondary postoperative hematoma after traumatic intracranial hematoma evacuation is a known complication, with highly varied reported incidence rate of 6.9–61%. (Bullock et al., 1990), (Richards and Hoff, 1974) We recently reported that alcohol inebriation at time of injury and hypocapnia during hospitalization were risk factors predicting recurrent hematoma after surgery for aSDH in the same cohort used in the present study (Heino et al., 2019). These results support previous reports showing that the administration of mannitol, alcohol intake, and coagulation profile influence recurrent hematoma rates after intracranial mass evacuation (Bullock et al., 1990). However, other factors such as the role of pre-injury anticoagulation/antithrombotic therapy remains controversial as to whether they truly impact postoperative hematoma rates (Palmer et al., 1994; Panczykowski and Okonkwo, 2011). Importantly, although commonly theorized to purport poor outcomes, reoperation after neurosurgical intervention has little supporting evidence suggesting a role in predicting outcomes (Palmer et al., 1994; Seifman et al., 2011; Lillemäe et al., 2017).

Despite that multiple predicting factors have been identified to be associated to outcome, it would be beneficial that studies could also focus towards differentiating causal or modifiable risk factors from mere predictive factors in aSDH outcomes. This distinction is

Vital for developing interventions that can genuinely improve patient outcomes. In addition, recent large study importantly highlights those variations in treatment strategies across different centers, namely the preference for acute surgical evacuation over conservative treatment, do not uniformly correlate with better functional outcomes (van Essen et al., 2022). These findings challenge prevailing assumptions about the superiority of aggressive surgical interventions in all aSDH cases and underscores the need for a more nuanced understanding of treatment efficacy and modifiable risk factors.

This study aims to explore potential associations between clinical outcomes and various factors, including postcraniotomy hematomas (PCHs), in patients with surgically treated acute subdural hematoma. It is essential to note that our analysis is observational, focusing on identifying associations rather than establishing any causality.

## 2 Methods

### 2.1 Study design

**Fig. 1:**
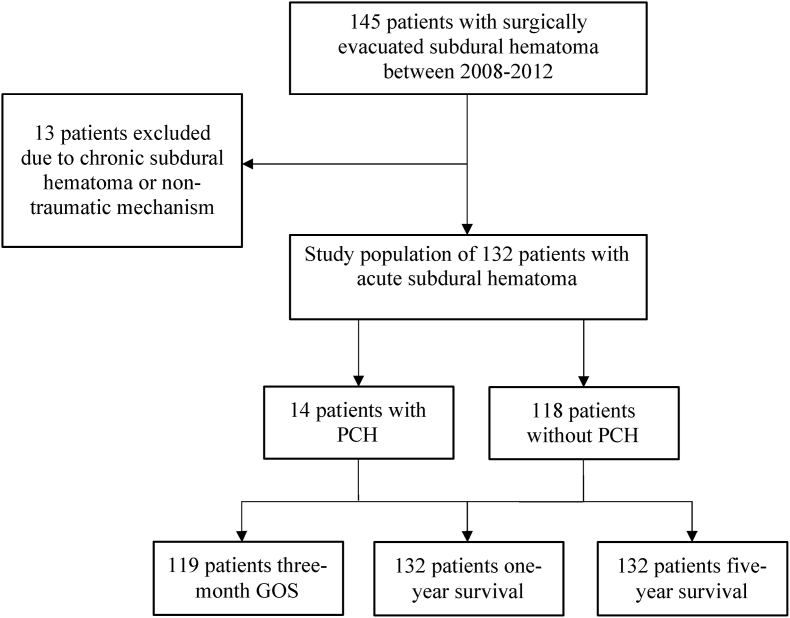
Flowchart of the study.

A review of the electronic database of the tertiary university hospital identified 145 consecutive patients between January 2008 and December 2012, after applying a search with International Statistical Classification of Diseases and Related Health Problems, 10th edition (ICD-10), code S06.5 and Nordic Classification of Surgical Procedure (NCSP) operation code AAD05. The inclusion criteria for this retrospective cohort study were traumatic mechanism causing the primary injury and an aSDH demonstrated on CT imaging. Additionally, surgical intervention with craniotomy in the acute period was required for the inclusion. PCH requiring surgical evacuation was defined as a newly expanding hematoma causing intracranial mass effect that was reoperated on within 30 days after primary evacuation. A total of 132 patients met these inclusion criteria including 14 who experienced PCH. The flow chart of the study summarizing the inclusion process is outlined in Fig. 1. The study was approved by the Institutional Review Board of the University Hospital. Patient consent was not required as this was a retrospective registry study. The study was conducted in accordance with the Declaration of Helsinki and its later amendments.

### 2.2 Study data and variables

After identifying the final 132 patients comprising the retrospective cohort both electronic and paper records of these patients were screened to collect pre-identified variables. In total, 165 variables were manually gathered and categorized. The dataset was divided into demographic, prehospital, preoperative, intraoperative and postoperative sections. The demographic features and prehospital data consisted of patient characteristics, pre-existing medications, smoking, the nature and mechanism of injury, the presence of other injuries, and other preoperative care data. Data on the level of consciousness was gathered at four time.

Points using GCS: at the trauma scene by paramedics, at hospital admission, preoperatively, and postoperatively. Pupil reactivity and documentation of neuroworsening of patients was also gathered. Neuroworsening was defined as a decrease of two or more points in the GCS motor score, or new loss of pupil reactivity or expanding subdural hematoma on CT. The AIS score was used to classify all the injuries after the trauma by each body region (Loftis et al., 2018). Laboratory data consisted of hematocrit, hemoglobin, platelet count, electrolytes, international normalized ratio, glucose, blood gas analysis, and blood alcohol level, which were gathered and categorized as lowest and highest values preoperatively, intraoperatively, and postoperatively. Blood alcohol level >0.05% was considered as a significant alcohol level. The intraoperative data was gathered from operation reports and consisted of operation time, surgeon experience, amount of intraoperative bleeding, intraoperative complications, and transfusions of packed red blood cells, fresh-frozen plasma, or platelets. Imaging data was collected from the CT imaging database. The thickness of a hematoma in the axial plane was manually measured by using an imaging software tool (Carestream Health, USA). Hematoma volumes were measured from the worst preoperative CT with a region of interest tool by calculating a manually drawn region of interest on each CT slice (mm^2^), multiplied by the slice thickness (5 mm). Compression of the basal cisterns and third ventricle was evaluated and midline shift was manually measured from the level of the third ventricle in axial plane (mm).

The Glasgow Outcome Scale (GOS) was used to grade functional outcome, and the evaluation of functional outcomes using the GOS was systematically conducted during a scheduled follow-up visit. This assessment was performed by either a qualified neurosurgeon or a trained resident, specifically 3 months post-injury. To ensure consistency and accuracy, the evaluation followed a structured approach, although it was not conducted through an official standardized interview format (Jennett and Bond, 1975). Outcome was categorized as unfavorable (GOS 1–3) or favorable (GOS 4–5). Time of death was collected from the patients’ electronical database to analyze survival rates at one and five years after the primary trauma.

### 2.3 Statistical analysis

All statistical analyses for this study were performed with SAS 9.4 (SAS Institute Inc., Cary, NC, USA). For the continuous variables, the Pearson correlation was first used to test the correlations of variables. Variables having a strong correlation (r > 0.70) were regarded as mathematically equivalent and only one of those were chosen for use in final models. A univariate logistic regression analysis was performed to define the factors related to the outcome and survival. Then, a multivariable logistic analysis was performed to identify

Independent factors to the functional outcome and survival. Odds ratios with 95% confidence interval (CI) were included in calculations. The receiver operating characteristic (ROC) curves with 95% confidence intervals (CIs) and areas under the curve (AUC) were calculated for multivariable models. The level of significance was set at α = 0.05.

## 3 Results

### 3.1 Study population

Demographic analysis showed a higher proportion of male patients in the cohort (102/132, 77.3%), while falls were the most common mechanism of injury (82.0 %) (Table 1). The average age of patients included in the cohort was 59.9 ± 15.4 years among patients without PCH and with PCH 50.7 ± 16.8 (OR, 0.97; 95% CI, 0.94–1.00; P = 0.04). Significant alcohol levels were detected in 43.4 % of cases and the proportion of smokers was 34.5 %. In addition, former abuse of alcohol was mentioned in the medical records of 69 patients (52.3 %). Neuroworsening state between the injury and the surgery occurred in 87 patients (65.9 %), while 22 patients (16.7 %) suffered from convulsions. The proportion patients taking anticoagulant or antiplatelet drugs prior to their aSDH was 12.9 % and 18.9 % respectively. The overall in-hospital mortality rate was 16.7 %.

**Table 1:** Basic characteristics comparing patients with and without postcraniotomy hematoma.

Characteristics	Patients without PCH	Patients with	Odds Ratio	P-value
	(n = 118)	PCH (n = 14)	(95% CI)	
Mean age, years ± SD	59.9 ± 15.4	50.7 ± 16.8	0.97 (0.94–1.00)	0.04
Sex (m/f)	91/27	11/3	0.92 (0.24–3.54)	0.90
Cause of injury^a^			1.44 (0.74–2.77)	0.28
Fall	95 (83.3%)	10 (71.4%)		
Traffic accident	9 (7.9%)	2 (14.3%)		
Assault	7 (6.1%)	1 (7.1%)		
Other	3 (2.6%)	1 (7.1%)		
Blood alcohol >0.5 ‰^b^	47 (40.9%)	9 (64.3%)	0.38 (0.12–1.22)	0.10
Smoking^c^	35 (33.7%)	5 (41.7%)	1.41 (0.42–4.76)	0.58
Use of anticoagulant or antiplatelet drug	38 (32.2%)	3 (21.4%)	0.57 (0.15–2.18)	0.41

PCH = postcraniotomy hematoma; CI = confidence interval; SD = standard deviation.

a No data for 4 patients without PCH.

b No data for 3 patients without PCH.

c No data for 2 patients with PCH and 14 patients without PCH.

### 3.2 Postcraniotomy hematoma and outcome

A total of 14 (10.6%) postcraniotomy hematomas were identified in the cohort (mean time in days between operations 6.2 ± 10.0). Seven of the 14 patients (50 %) had an unfavorable outcome (GOS 1–3) while three patients (21%) had a favorable outcome (GOS 4–5) at three months. Four patients had no follow-up visit, and their GOS at three months was unknown. There was no statistical difference in three-month GOS between PCH and non- PCH group (p = 0.37).

After one year 11 out of 14 (79 %) patients with PCH were alive. Of the patients who underwent only initial evacuation without hematoma recurrence, 65 of 118 patients (55 %) were alive at one year. The five-year survival rate amongst the patients with PCH was 57 % (=8 patients), while in those without PCH the proportion was 55 of 118 (47 %). Between PCH and non-PCH groups there was no significant difference in survival after one year (p = 0.34) or five years (p = 0.37). (Table 2).

**Table 2:** Characteristics, outcome and survival of patients with PCH.

Patient	Anticoagulant or antiplatelet	Primary hematoma volume mm^3^	Other intracranial lesions	PCH type	3-month GOS	Survival 1 year	Survival 5 years
60 yr old female	Warfarin	47	Contusion	SDH	ND	Yes	No
39 yr old male	None	80	Cortical SAH	SDH	1	No	No
23 yr old male	None	25	EDH	EDH	4	Yes	Yes
58 yr old male	None	114	ICH	ICH	1	No	No
35 yr old male	None	40	Contusion, cortical SAH	SDH	4	Yes	Yes
61 yr old male	None	95	Contusion	SDH	3	Yes	Yes
44 yr old male	None	17	ICH, cortical SAH	ICH	3	Yes	Yes
51 yr old male	None	81	None	SDH	ND	Yes	No
72 yr old male	Aspirin	45	ICH, cortical and basal SAH	ICH	1	No	No
46 yr old female	None	15	Contusion	SDH + EDH	ND	Yes	Yes
21 yr old male	None	68	None	SDH + ICH	4	Yes	Yes
75 yr old male	Warfarin	128	None	SDH + EDH	3	Yes	No
63 yr old male	None	195	Contusion	SDH + EDH	3	Yes	Yes
62 yr old female	None	58	None	SDH + EDH	ND	Yes	Yes
					p = 0.37	p = 0.34	p = 0.37
					OR 0.53 (0.13–2.14)	OR 1.98 (0.52–9.54)	OR 1.84 (0.50–7.53)

EDH = Epidural hematoma; GOS = Glasgow outcome scale; ICH = Intracerebral hemorrhage; PCH = Postcraniotomy hematoma; ND = No data; OR = Odds ratio; SAH = Subarachnoid hemorrhage; SDH = Subdural hematoma; OR = odds ratio; (.) = 95% confidence interval.

### 3.3 Factors associated with three-month outcome

Outcome data was available for 119 of 132 patients (90.2 %). Of these, 67 patients (56.3 %) had an unfavorable outcome (GOS 1–3) and 52 patients (43.7 %) had a.

Favorable outcome (GOS 4–5). Mean hematoma volume in patients with unfavorable outcome was 18.9 mm^3^ larger than in those with favorable outcome (98.4 vs. 79.5 mm^3^ respectively, p. = 0.044). The mean AIS score was 1.2 points higher in the unfavorable vs. favorable patients (8.3 vs. 7.1 respectively, p = 0.040). (Table 3).

**Table 3:** Factors associated with outcome and survival in univariate analysis.

3-month GOS			
	Favorable (GOS 4–5, n = 52)	Unfavorable (GOS 1–3, n = 67)	p-value
AIS score ± SD	7.1 ± 2.8	8.3 ± 3.5	0.040
Hematoma volume (mm^3^) ± SD	79.5 ± 40.6	98.4 ± 45.8	0.044

Survival at 1 year			
	Yes (n = 76)	No (n = 56)	p-value
Age (Year) ± SD	55.7 ± 15.9	63.4 ± 14.5	0.007
Antiplatelet use	9 (11.8 %)	16 (28.6 %)	0.018
GCS at the scene (IQR)^a^	14 (7–15)	9 (3–13)	<0.001
GCS at the arrival (IQR)^a^	12 (5–14.75)	7 (3–11.75)	0.017
Hematoma thickness (mm) ± SD	14.9 ± 5.7	17.3 ± 6.6	0.034
Smoking	29 (44.6 %)	11 (21.6 %)	0.011
Worsening of the neurological state	44 (57.9 %)	43 (76.8 %)	0.025

Survival at 5 years			
	Yes (n = 63)	No (n = 69)	p-value
Age (Year) ± SD	54.0 ± 16.5	63.4 ± 13.6	0.011
Antiplatelet use	7 (11.1 %)	18 (26.1 %)	0.033
Blood alcohol level (‰) ± SD	1.3 ± 1.6	0.8 ± 1.2	0.030
GCS at the scene (IQR)^a^	11 (5–14)	8 (3.5–13)	0.037
Hematoma volume (mm^3^) ± SD	82.1 ± 42.1	97.3 ± 44.7	0.049

AIS = Abbreviated Injury Scale; GCS = Glasgow Coma Scale; GOS = Glasgow Outcome Scale; SD = Standard deviation; IQR = Interquartile range.

a Pearson correlation >0.70 (r = 0.76).

The multivariable model identified both initial hematoma volume (OR 0.99, 95% CI 0.98–1.00, p = 0.009) and AIS score (OR 0.85, 95% CI 0.73–0.96, p = 0.016) as

**Fig. 2:**
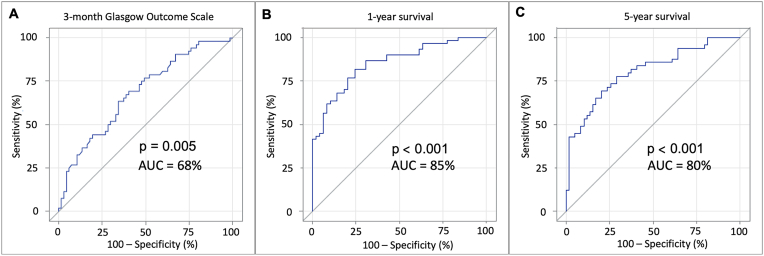
Receiver operating characteristic (ROC) curves for multivariable analysis. **(A)** ROC curve for multivariable analysis of three-month Glasgow Outcome Scale score (Area Under the Curve (AUC) = 0.68, 95% CI 0.59–0.78, p = 0.005). The multivariable model identified the volume of hematoma (OR 0.99, 95% CI 0.98–1.00, p = 0.009) and the AIS score (OR 0.85, 95% CI 0.73–0.96, p = 0.016) as independent predictors for three-month outcome. **(B)** A ROC curve for the multivariable analysis of one-year survival (AUC = 0.85, 95% CI 0.78–0.92, p < 0.001). The multivariable model identified the pre-injury use of antiplatelet drug (OR 0.24, 95% CI 0.06–0.84, p = 0.030), worse GCS at the scene (OR 1.38, 95% CI 1.21–1.61, p < 0.001), neuroworsening (OR 0.16, 95% CI 0.04–0.54, p = 0.005), and increased age (OR 0.96, 95% CI 0.93–1.00, p = 0.048) as independent predictors for worse survival during the first year after injury. The proportion of smokers was significantly lower among deceased patients after the first year (OR 3.72, 95% CI 1.23–12.67, p = 0.026) **(C)** ROC curve for multivariable analysis of five-year survival (AUC = 0.80, 95% CI 0.72–0.89, p < 0.001). GOS at the scene (OR 1.25, 95% CI 1.12–1.41, p < 0.001), higher age (OR 0.95, 95% CI 0.92–0.98, p = 0.003), and blood alcohol on admission (OR 1.56, 95% CI 1.07–2.34, p = 0.025) were recognized as independent predictors for worse five-year survival.

Independent predictors for favorable vs. unfavorable outcome. The ROC AUC for the model was 0.68 (95% CI 0.59–0.78, p = 0.005). (Table 4 and Fig. 2).

**Table 4:** Factors associated with outcome and survival in multivariable logistic analysis.

3-month GOS				
	Estimate	S.E.	OR (95% CI)	p-value
AIS score	−0.17	0.07	0.85 (0.73–0.96)	**0.016**
Hematoma volume (mm^3^)	−0.01	<0.01	0.99 (0.98–1.00)	**0.009**
**Survival at 1 year**				
Age (Year)	−0.04	0.02	0.96 (0.93–1.00)	**0.048**
Antiplatelet use	−1.41	0.65	0.24 (0.06–0.84)	**0.030**
GCS at the scene	0.32	0.07	1.38 (1.21–1.61)	**<0.001**
Hematoma thickness (mm)	−0.04	0.04	0.96 (0.88–1.04)	0.307
Smoking	1.31	0.59	3.72 (1.23–12.67)	**0.026**
Worsening of the neurological state	−1.82	0.66	0.16 (0.04–0.54)	**0.005**
**Survival at 5 years**				
Age (Year)	−0.05	0.02	0.95 (0.92–0.98)	**0.003**
Antiplatelet use	−0.36	0.63	0.70 (0.19–2.36)	0.570
Blood alcohol level (‰)	0.44	0.20	1.56 (1.07–2.34)	**0.025**
GCS at the scene	0.22	0.06	1.25 (1.12–1.41)	**<0.001**
Hematoma volume (mm^3^)	>-0.01	0.01	1.00 (0.99–1.01)	0.523

P-values of the independent predictors are bolded.

AIS = Abbreviated Injury Scale; CI = Confidence interval; GCS = Glasgow Coma Scale; GOS = Glasgow Outcome Scale; OR = Odds Ratio; S.E. = Standard error.

## 4 Factors associated with survival

After the first year the overall survival rate was 57.6% (76 of 132 patients). Among those alive at one-year nine patients were using an antiplatelet agent prior to the aSDH (p = 0.018) and neurological decline occurred in 44 of the 76 patients (p = 0.025). Both GCS at the scene and at arrival were significantly higher in survivors (GCS score 14 (7–15) vs. 10 (3–14.5), p < 0.001, and 11 (5–15) vs. 7 (3–12), p = 0.017, respectively). Because the Pearson correlation between these was strong (r = 0.76, p < 0.05), GCS at the scene was used for the multivariable analysis. Additional significant characteristics of survivors included lower thickness of initial hematoma (14.9 vs. 17.3 mm, p = 0.034), younger age (55.7 vs. 63.4 years, p = 0.007), and higher chance of being a smoker (29 vs. 11 patients, p = 0.011). (Table 3).

The multivariable model identified pre-injury use of an antiplatelet agent (OR 0.24, 95% CI 0.06–0.84, p = 0.030), worse GCS at the scene (OR 1.38, 95% CI 1.21–1.61, p < 0.001), neurological decline (OR 0.16, 95% CI 0.04–0.54, p = 0.005), and higher age (OR 0.96, 95% CI 0.93–1.00, p = 0.048) as independent predictors for mortality at one year following injury. Contrastingly, the proportion of smokers was significantly lower among deceased after the first year (OR 3.72, 95% CI 1.23–12.67, p = 0.026). The ROC AUC for the multivariable model was 0.85 (95% CI 0.78–0.92, p < 0.001) (Table 4 and Fig. 2).

The five-year survival rate was 47.7% (63 of 132 patients). Smaller hematoma volume (82.1 vs. 97.3 mm^3^, p = 0.049), lower chance of using antiplatelets prior to the event (11.1 vs. 26.1 % respectively, p = 0.033), higher GCS at the scene (11 vs. 8, p = 0.037), higher likelihood of having high blood alcohol on admission (1.32 vs. 0.76 ‰, p = 0.030), and lower age at the time of injury (54.0 vs. 63.4 years, p = 0.001) were found amongst patients who were alive at five years in comparison to those who were not (Table 2).

After performing a multivariable logistic regression lower GCS score at the scene (OR 1.25, 95% CI 1.12–1.41, p < 0.001) and higher age (OR 0.95, 95% CI 0.92–0.98, p = 0.003) were independent predictors for decreased five-year survival. In addition, higher likelihood of having a high blood alcohol level on admission (OR 1.56, 95% CI 1.07–2.34, p = 0.025) was identified in the multivariable logistic model as a significant factor. The ROC AUC for the model was 0.80 (95% CI 0.72–0.89, p < 0.001) (Table 4 and Fig. 2).

## 5 Discussion

Postoperative hematoma following an intracranial intervention has been theorized without substantive evidence to be associated with significant morbidity and mortality, although the definition of this term in different studies has varied significantly. (Seifman et al., 2011; Ito et al., 1976), (Mellergård and Wisten, 1996) The inconsistency in the definition of a postoperative hematoma has previously been broad, encompassing various factors such as the initial settings of surgical intervention, indications for surgery, and the types of surgical techniques employed. Thus, the resulting comparisons between different studies have proven to be difficult. As a result, both the incidence of PCH after evacuation of a traumatic mass lesion has widely varied in earlier literature and is unclear, as well as its association with outcomes. Contrastingly, previous studies have routinely identified factors such factors as age, hypertension, anticoagulant/antiplatelet use, and alcohol intake to associate with PCH formation (Bullock et al., 1990; Kalfas and Little, 1988; Basali et al., 2000). Therefore, although previous literature has substantially documented certain risk factors impacting PCH formation, the association between PCH and mortality has not been thoroughly substantiated prior to this study (Chrastina et al., 2020; Bullock et al., 1990; Su et al., 2008). Here, we report that PCH in fact may not have a significant impact on either functional outcome as measured by GOS or survival following surgical intervention.

### 5.1 Postcraniotomy hematoma

We found no association between functional outcome or mortality and PCH after aSDH evacuation. This finding seems discordant with some earlier findings suggesting the correlation between PCH and worse outcome and increased mortality after surgery for traumatic intracranial mass lesions. Bullock et al. (1990) recognized worse outcome among PCH patients requiring secondary operation in their study of 850 patients (Bullock et al., 1990).

Therefore, although our initial result may appear discordant with previous analyses, this may be explained by several factors. The role of neurocritical care is vital enabling more favorable recovery in these patients, and has undergone substantial advances in the recent decades (Stocchetti et al., 2017). Advanced cardiovascular monitoring, including echocardiography and intravascular volume assessment, give opportunities to avoid harmful changes in cerebral blood

Flow whether that be volume depletion or overload leading to changes in intracranial pressure that could influence these outcomes (Stocchetti et al., 2017; Le Roux et al., 2014). As has been demonstrated previously, the rapid diagnosis of rebleeding and early surgical intervention has also been linked with better outcomes in the setting of aSDH post-operative complications (Lillemäe et al., 2017; Carney et al., 2017; Amiri et al., 2013; Lawton et al., 1995). Given advanced neuromonitoring, increased availability of rapid neuroimaging, and additional advances over the past decade, it is plausible that the diagnosis of rebleeding and subsequent surgical intervention may often occur at an earlier interval, thus changing these patient's outcomes.

### 5.2 Factors associated to outcome and survival

Earlier studies have reported a correlation between worse outcome and several other factors besides PCH after aSDH such as GCS score, pupillary reactivity, concurrent brain lesions, and other CT findings (Hatashita et al., 1993; Servadei et al., 2000; Sakas et al., 1995; Hlatky et al., 2007). Foreman et al. (2007) reported a correlation between TBI 12-month outcome and GCS score, AIS, and Injury Severity Scale (ISS) (Foreman et al., 2007). Another prospective study identified GCS score, age, pupillary reactivity, ISS and presence of hematoma as independent predictors of one-year survival after TBI (Signorini et al., 1999). One limitation to these studies, however, was that they included patients other than those with aSDH limiting the specificity of these findings to the aSDH population. In light of this, we also found in this specific population that GCS score was an independent predictor of survival after one year and five years, which supports previous studies that had broader inclusion criteria. In addition, the higher AIS score was a risk factor for the worse 3-month outcome in this setting. The correlation between AIS and a discharge destination has been reported in pediatric population, as well (Hubbard et al., 2019). However, there was no correlation between pupillary reactivity on outcome or survival in this study, which was not the finding in the validated prognostic models IMPACT and CRASH studies, strongly driven by pupillary reactions. Notably, Bullock et al. (2006) also defined indications for surgery of aSDH including loss of pupillary reactivity as an indication (Bullock et al., 2006). As a result, patients with declining GCS score or pupillary abnormalities are generally considered to warrant a surgical evacuation of the hematoma, which are indications broadly followed at many institutions. Notably, in this present cohort, neurological decline was also a risk factor for mortality during the first year.

Recent evidence, including analyses from the van Essen et al., 2022 challenges the previously assumed consensus in the neurosurgical community regarding CT findings that dictate surgical intervention in aSDH (Bullock et al., 2006; van Essen et al., 2022). That study highlight substantial variability in practice and indicate that outcomes do not significantly differ between centers with more aggressive or conservative surgical policies (van Essen et al., 2022). Our traditional assertion about the standard of

Midline shift greater than 5 mm and clot thickness greater than 10 mm as clear indicators for hematoma evacuation must therefore be re-evaluated in light of these findings. Recognizing this, our study's focus on aSDH patients selected for surgery may introduce bias in analyzing outcome-associated variables. A more comprehensive approach would involve examining all aSDH patients, with surgery as a predictive variable, to provide a broader understanding of the various factors influencing outcomes in these cases.

Other studies have suggested that the thickness of the hematoma also influences the outcome (Zumkeller et al., 1996; Servadei et al., 2000). We also found a significant correlation between the thickness of the hematoma and one-year survival, although when controlling for other variables, it was not identified as an independent predictor in the multivariable analysis. While earlier studies have identified the degree of the midline shift as a factor correlating with poorer outcome and mortality, we did not find this interaction in our specific population (Kotwica and Brzeziński, 1993; Howard et al., 1989; Moussa et al., 2018; Bartels et al., 2015). In our study, the mean midline shift was over 2 mm greater among patients with a poor outcome, but this was not a statistically significant difference between groups.

Hematoma volume as a predictor for outcome after aSDH is a more controversial topic in contrast. Howard et al. (1989) initially reported a correlation between larger aSDH volume and poorer outcome (Howard et al., 1989). Contrastingly, van den Brink et al. (1999) reported no correlation between these factors in multivariable analyses in a cohort including patients with either epidural hematomas and aSDHs (van den Brink et al., 1999). Our findings support the results of Howard et al. (1989), given that in the present study a 25% larger hematoma volume was found among those with unfavorable outcome.

Age is another known key predictor for outcome after TBI (Hukkelhoven et al., 2003; Flaada et al., 2007). Wilberger et al. (1991) reported that patients with aSDH aged over 65 years had a worse outcome compared to their younger compatriots (Wilberger et al., 1991). However, age alone was not an independent predictor for outcome in a prior multivariable analysis in patients with aSDH that underwent surgical evacuation (Seelig et al., 1981). We identified age as an independent predictor of survival after one and five years in our multivariable analysis. However, there was no correlation between age and three-month functional outcome in this cohort.

Although present knowledge suggests increased risk of SDH among those using antiplatelet or anticoagulant medications, the impact on functional outcome and survival is less well described (Wintzen and Tijssen, 1982; Gaist et al., 2017). As previously mentioned, as the population ages the prevalence of these medications is increasing and therefore predisposing more patients to intracranial bleeding. Post-traumatic ICH has been associated with increased mortality in patients with antiplatelet therapy (Gaist et al., 2017; Mina et al., 2002). In addition, Jones et al. (2006) recognized that patients with former use of clopidogrel treatment were at higher risk for neurosurgical interventions, rebleeding and greater need for blood product transfusions (Jones et al., 2006). Wong et al. (2008) also reported that discharging to the long-term inpatient facility was more common among those receiving clopidogrel therapy prior to their injury (Wong et al., 2008). In contrast, Bachelani et al. (2011) did not report a significant association between aspirin use and mortality, outcome, progression of ICH, or craniotomy in patients with ICH (Bachelani et al., 2011). Here we add that antiplatelet use was an independent predictor for worse survival after five years. There was a correlation to survival after one year, but multivariable analysis recognized no significant predictive value. Additionally, neither antiplatelet nor anticoagulant use had a significant impact on three-month outcomes. One notable fact is that the proportion of anticoagulant users was relatively small (12.9%) in this study suggesting that the total effect of anticoagulant therapy may be underpowered.

Interestingly, we found that smoking was associated with improved survival at one year following aSDH. This finding is supported by preclinical animal models both *in vitro* and *in vivo* studies showing neuroprotective features of nicotine (Akaike et al., 1994; Owman et al., 1989). Similarly, up-regulation of nicotinic acetylcholine receptors (nAChRs) has been noticed among smokers and an important role of these receptors in cognitive functions has been reported (Mansvelder and McGehee, 2002; Changeux et al., 1998). Östberg and Tenovuo found no correlation between smoking and outcome after TBI in their prospective study with 689 patients (Ostberg and Tenovuo, 2014). Therefore, it appears that smoking may has some impact on outcome, but our study is purely observational and does not provide any causality. Thus, this controversy topic remains to be studied other type of research settings.

We also found an association between presence of alcohol at time of admission with better five-year survival. The consumption of alcohol is a well-known risk factor for TBI (Albrecht et al., 2018). However, Tien et al. reported protective effects of low to moderate level of blood alcohol for outcome after severe TBI (Tien et al., 2006). In a meta-analysis of observational studies with a total of 95,941 patients, Raj et al. found an association between blood alcohol and decreased risk of death (Raj et al., 2016). In addition, animal studies have indicated a neuroprotective value of low to moderate doses of alcohol after TBI (Kelly et al., 2000; Gottesfeld et al., 2002). However, other studies have been unable to replicate these

Protective effects (Jurkovich et al., 1993; Chen et al., 2012). Moreover, earlier studies have focused on in-hospital mortality and there is limited data about the effects of alcohol to the later mortality, suggesting a need for future

Studies to verify this finding. Similarly as above, our study was just able to show an association, and this study does not provide any causality. More studies are needed to study this intriguing and controversy topic further.

### 5.3 Limitations

This study has several limitations that should be considered when interpreting these results. The retrospective study design inherently comes with missing data, including prehospital factors and factors that occur after discharge. Our study population was also relatively small when dividing into subgroups for analysis. Therefore, it may be that we were not able to detect all possible associations between potential predictive factors and outcome due to insufficient power. In addition, some patients may not have undergone surgery after primary aSDH or PCH due to a clinical decision made by the neurosurgeon or family. However, a consecutive patient selection was used to avoid interindividual variation within the hospital. The actual cause of death was not available for most patients and therefore, there is no possible way to assess causal relationship between the explored factors and cause of death. In addition, our study design does not prove causality given its retrospective observational nature. Thus, these results should be treated purely as predictive associations and further studies with greater population size and a prospective study setting are necessary to verify or dismiss these findings.

## 6 Conclusion

The occurrence of PCH requiring reoperation was not associated with outcome or survival in this study. Larger hematoma volume and higher AIS score were independent predictive factors for worse three-month functional outcome after surgery for aSDH, while a lower GCS score at the trauma scene and increased age were identified as predictors for worse survival after one and five years. In addition, use of antiplatelet drugs and neurological decline between the trauma event and surgery were predictors for worse survival after one year. Smoking was associated with survival at one year and higher alcohol with survival at five years. This study adds important information to understanding contributing factors to postoperative death and functional outcome after a common neurosurgical problem of aSDH, and suggests that active monitoring and immediate surgical interventions are necessary and beneficial. Also, it is important to note that additional prospective and larger retrospective studies are required to provide further insight into these results since any causality was not shown in this study.

## Funding

This work was supported by grants from the 10.13039/501100007639Maire Taponen foundation, Sigrid Juselius Foundation, 10.13039/100010129Maud Kuistila Foundation, 10.13039/100010135Finnish Medical Association to JK, by 10.13039/501100006306Sigrid Juselius Foundation to IH, by the 10.13039/501100007639Maire Taponen foundation and 10.13039/501100006306Sigrid Juselius Foundation to AS, by the 10.13039/501100002341Academy of Finland (#17379), Government's Special Financial Transfer tied to academic research in Health Sciences, Finland (#11123 and #11129) and the 10.13039/501100007639Maire Taponen Foundation to JPP.

## Ethical approval

The study was approved by the Institutional Review Board of the University Hospital. Patient consent was not required as this was a retrospective registry study. Thus, the need of patient consent was waived by the Institutional Review Board. All procedures performed in studies involving human participants were in accordance with the ethical standards of the 1964 Helsinki declaration and its later amendments or comparable ethical standards.

## Author contributions

J.K, J.F., O.T. and J.R. devised this study. I.H, J.K. and J.F. designed the study protocol. All the data of patients were collected by I.H, A.S. and J.K. The data was analyzed by Y.C (biostatistician), J.K, I.H and A.S. The first versions of the manuscript were drafted by I.H, J.F, A.S., S.B.L. and J.K. with critical contributions from J.R, R.G, A.J.K, A.J.S, S.H, I.S, D. L, Y.C, J.F.R, J.P.P, R.S.K.T, A.S. and O.T. All authors reviewed, edited and approved the final version. All authors agreed J.K. to act as a corresponding author.

## Data availability statement

Fully anonymized data relevant to this study will be shared by requesting it from the corresponding author. Appropriate Institutional Review Board approvals and research qualifications are required.

## Declaration of competing interest

The authors declare the following financial interests/personal relationships which may be considered as potential competing interests:Janne Koskimaki reports financial support was provided by Maire Taponen Foundation. Janne Koskimaki reports financial support was provided by Sigrid Jusélius Foundation. Janne Koskimaki reports financial support was provided by 10.13039/100010135Finnish Medical Association. Janne Koskimaki reports financial support was provided by 10.13039/100010129Maud Kuistila Memorial Foundation. Iiro Heino reports financial support was provided by Sigrid Jusélius Foundation. Antti Sajanti reports financial support was provided by Sigrid Jusélius Foundation. Antti Sajanti reports financial support was provided by Maire Taponen Foundation. Jussi Posti reports financial support was provided by 10.13039/501100002341Academy of Finland.
